# Bioenergetic Dysfunction and Inflammation in Alzheimer’s Disease: A Possible Connection

**DOI:** 10.3389/fnagi.2014.00311

**Published:** 2014-11-10

**Authors:** Heather M. Wilkins, Steven M. Carl, Alison C. S. Greenlief, Barry W. Festoff, Russell H. Swerdlow

**Affiliations:** ^1^Department of Neurology, University of Kansas Medical Center, Kansas City, KS, USA; ^2^University of Kansas Alzheimer’s Disease Center, University of Kansas Medical Center, Kansas City, KS, USA; ^3^Department of Pharmacology, University of Kansas Medical Center, Kansas City, KS, USA; ^4^Department of Molecular and Integrative Physiology, University of Kansas Medical Center, Kansas City, KS, USA; ^5^pHLOGISTIX Neurodiagnostics, Lenexa, KS, USA; ^6^Department of Biochemistry and Molecular Biology, University of Kansas Medical Center, Kansas City, KS, USA

**Keywords:** inflammation, bioenergetics, DAMP, mitochondria, Alzheimer’s disease

## Abstract

Inflammation is observed in Alzheimer’s disease (AD) subject brains. Inflammation-relevant genes are increasingly implicated in AD genetic studies, and inflammatory cytokines to some extent even function as peripheral biomarkers. What underlies AD inflammation is unclear, but no “foreign” agent has been implicated. This suggests that internally produced damage-associated molecular pattern (DAMPs) molecules may drive inflammation in AD. A more complete characterization and understanding of AD-relevant DAMPs could advance our understanding of AD and suggest novel therapeutic strategies. In this review, we consider the possibility that mitochondria, intracellular organelles that resemble bacteria in many ways, trigger and maintain chronic inflammation in AD subjects. Data supporting the possible nexus between AD-associated bioenergetic dysfunction are discussed.

## Inflammation in AD

The role of inflammation in Alzheimer’s disease (AD) was first observed approximately four decades ago (Ishii and Haga, 1976). Beyond the identification of elevated immune cells and cytokines in AD brain, many lines of evidence implicate inflammation as a pathological AD hallmark. For instance, the use of non-steroidal anti-inflammatory drugs (NSAIDs) associates with decreased AD risk (Breitner et al., 1994; Rich et al., 1995). Additionally, a number of genes recently associated with AD risk through genome wide association studies (GWAS) play a role in inflammation or inflammatory signaling.

While an immediate response to an invasive pathogen is beneficial, a sustained inflammatory reaction will lead to tissue damage and functional decline. The cells responsible for an immune response in the brain are microglia and astrocytes (Akiyama et al., 2000a). In 1994, Akiyama et al. reviewed the contribution of microglial activation to neuroinflammation in AD. The β-amyloid plaques of deceased AD patients were found to contain significant amounts of activated microglia (Akiyama, 1994). Microglia from AD patients express major histocompatibility complex class II (MHCII) molecules, cyclooxygenase 2 (COX2), and cytokines/chemokines such as monocyte chemotactic protein 1 (MCP-1), TNFα, and IL-1β (Akiyama et al., 2000b). Postmortem AD brain analysis depicted elevated IL-1α, CXCR2 (IL-8 receptor β), CCR3 (C-C chemokine receptor type 3, CD193), CCR5 (C-C chemokine receptor type 5, CD195), and transforming growth factor β (TGFβ) (Cartier et al., 2005). The activity of numerous immune system pathways are increased in AD, including complement, membrane attack complexes (MAC), and cytokines such as; IL-1, IL-6, and TNFα. These have been extensively reviewed elsewhere (Akiyama et al., 2000a).

Beyond direct measurement of inflammatory pathway mediators and effectors, brain inflammation can be measured in living subjects through the use of PK11195. This molecule is an isoquinoline carboxamide, which binds to the peripheral benzodiazepine receptor. Within the context of the central nervous system (CNS), PK11195 binds to glial cells, such as astrocytes and microglia. Compared to control subjects, AD patients have elevated binding of radiolabeled PK11195. Furthermore, a correlation between brain PK11195 labeling and cognitive deterioration is prominent in AD patients (Cagnin et al., 2001; Versijpt et al., 2003).

Recent genetic association studies have revealed genes that regulate or encode inflammatory proteins associated with AD risk. These are reviewed here and in Table 1. Two genes, which specifically regulate phagocytosis influence AD risk. R47H, a novel genetic variant of triggering receptor expressed on myeloid cells 2 (TREM2), impedes the normal function of this protein (Jonsson et al., 2013). TREM2 functions to suppress cytokine production and modulates microglial phenotype into a more phagocytic role. Mutations of TREM2 reduce microglial phagocytosis by preventing its maturation and transport to the cell membrane (Kleinberger et al., 2014). Cerebral spinal fluid (CSF) levels of soluble TREM2 are reduced in AD subjects, although no significant difference is observed in plasma (Kleinberger et al., 2014). A potential role for TREM2 in AD has been reviewed extensively elsewhere (Jiang et al., 2013). The other gene, adenosine triphosphate (ATP)-binding cassette subtype family A member 7 (ABCA7), encodes a protein, which regulates macrophage phagocytosis and the transport of molecules across the plasma membrane (Hollingworth et al., 2011).

**Table 1:** **Genetic variants associated with AD risk and inflammatory pathways**.

Gene symbol	Function	Variation associated with AD	Reference
TREM2	Phagocytosis/control of microglial phenotype	rs75932628 (R47H)	(Jiang et al., 2013; Jonsson et al., 2013; Kleinberger et al., 2014)
CR1	Complement signaling phagocytosis	rs6656401	(Lambert et al., 2009; Hazrati et al., 2012)
EPHA1	Epithelial cell barriers	rs11767557	(Hollingworth et al., 2011)
	Cell motility and morphology	rs11771145	
HLA-DRB5/DRB1	MHC signaling	rs9271172	(Lambert et al., 2013)
INPPD5	Microglial cell function	rs35349669	(Lambert et al., 2013)
MEFC2	Effector of MAPK signaling/activates c-Jun	rs190982	(Lambert et al., 2013)
PTK2B	Activates MAPK signaling	rs28834970	(Lambert et al., 2013)
CD33	Cell–cell interactions	rs3865444	(Hollingworth et al., 2011)
	Immune cell function endocytosis	rs3826656	
ABCA7	Transporter	rs3764650	(Hollingworth et al., 2011)
	Phagocytosis	rs3752246	
CLU	Complement NFκB signaling	rs11136000	(Harold et al., 2009; Lambert et al., 2009)

Single nucleotide polymorphisms of genes encoding components of the complement cascade and MHC molecules enhance AD risk. The complement system aids in antibody presentation and phagocytosis processes relevant to the clearance of antigens or inflammatory stimuli (Hazrati et al., 2012). Complement component receptor 1 (CR1) and clusterin (CLU) associate with increased AD risk (Lambert et al., 2009). CR1 is a type one transmembrane protein involved in activation of glial cells. CR1 activates complement signaling and phagocytosis (Lambert et al., 2009). CLU is a complement inhibitor, with other functions that relate to apoptosis and modulation of NFκB signaling (Harold et al., 2009; Lambert et al., 2009). MHC molecules present antigens for immune responses, of which one single nucleotide polymorphisms (SNP) found in the HLA-DRB5/DRB1 gene is a risk factor for AD.

Genes encoding proteins, which regulate immune cell function (EPHA1, INPPD5, and CD33) associate with AD risk. Ephrin type A receptor 1 (EPHA1) is highly expressed in early stages of inflammation and may play a role in the redistribution of epithelial cell barriers (Ivanov and Romanovsky, 2006). EPHA1 also regulates cell motility and morphology. Phosphatidylinositol-3,4,5-trisphosphate 5-phosphatase 1 (INPPD5) is expressed in hematopoietic cells (which give rise to immune cells such as macrophages and T cells). INPPD5 has been implicated in the regulation of microglial cell function. CD33 is a transmembrane receptor, which belongs to the Siglec family (sialic acid binding immunoglobulin-type lectins, or carbohydrate binding proteins), which bind and recognize sialic acid. Sialic acid is a general term for derivatives of neuraminic acid or the keto-deoxynonulosonic acid of nine-carbon sugars found on many proteins and lipids. Sialic acids function to mediate selective cell-to-cell interactions (Crocker and Varki, 2001). In particular, CD33 is expressed on myeloid cells where it promotes cell-to-cell interactions and regulates inflammatory function (Hollingworth et al., 2011).

Single nucleotide polymorphisms which alter inflammatory signaling pathways increase AD risk. Monocyte specific enhancing factor 2 (MEFC2) is a transcription factor. Protein tyrosine kinase 2β (PTK2B), is a cytoplasmic tyrosine kinase. PTK2B activates p38 MAPK signaling, which ultimately activates inflammatory pathways, while MEFC2 is a downstream effector of p38 MAPK signaling (Han et al., 1997). C-Jun activation is mediated by MEFC2, and is thus highly associated with inflammatory cascades. Notably, c-Jun is up-regulated in hippocampi from AD subject postmortem brain tissue (Marcus et al., 1998). Genetic susceptibility loci and polymorphisms for each of these genes have been associated with AD risk (McGeer and McGeer, 2001; Ivanov and Romanovsky, 2006; Hollingworth et al., 2011; Hazrati et al., 2012; Lambert et al., 2013).

To some extent peripheral inflammatory markers may reflect one’s likelihood of developing dementia. Some conflict exists regarding IL-6 and C reactive protein (CRP) correlation with disease risk. While IL-6 and CRP levels positively correlate with dementia risk, other studies found no association with AD risk (Engelhart et al., 2004; Sundelof et al., 2009). A relationship between IL-1, TNFα, and the development of AD has been observed (Tan et al., 2007). In one eloquent longitudinal study, midlife IL-6 elevation served as a predictor of cognitive decline (Singh-Manoux et al., 2014). Thus, not only are plasma inflammatory markers promising early biomarkers, inflammation-related changes appear to precede the overt clinical dementia phenotype.

Strong evidence linking neuroinflammation and AD progression has propagated a new hypothesis termed “cycle of self-perpetuating inflammatory neurotoxicity.” This hypothesis suggests that after the initial inflammatory stimuli activates microglia, the inflammatory tissue damage induces neuronal cell death that ultimately propagates to other brain areas (Block et al., 2007). Chronic inflammation is proposed to be a result of two possible factors; a lack of clearance of the inflammatory stimulus, or a failure to resolve the inflammatory process (Glass et al., 2010). It is further hypothesized that a disruption of homeostasis between cell death and phagocytosis is a prominent factor contributing to pathological inflammation in diseases (Zitvogel et al., 2010).

The factor or factors that initiate inflammation in AD remain elusive. One factor that may substantially contribute to chronic inflammation is bioenergetic dysfunction, which may arise at the level of the mitochondria. We review the evidence for bioenergetic dysfunction in AD below.

## Bioenergetic Dysfunction in AD

The term “bioenergetics” refers to cell energy metabolism. Bioenergetic flux is observed through the overall flow of individual biochemical pathways (Swerdlow, 2014). Three major bioenergetic pathways facilitate carbohydrate-based energy production – glycolysis, the Krebs cycle (or the citric acid cycle; TCA), and oxidative phosphorylation. These pathways are commonly examined when determining bioenergetic function. Glycolysis is an anaerobic process that takes place in the cytoplasm and results in low energy yield. The aerobic TCA cycle and oxidative phosphorylation phases, which occur at the matrix and inner membrane of mitochondria, respectively, can generate larger amounts of energy. Bioenergetic flux is a multidirectional measurement, with forward movement referring to the energy producing, catabolic direction (i.e., glycolysis → TCA → oxidative phosphorylation). The brain is particularly vulnerable to reductions in aerobic bioenergetic flux because of its high energy demand and relatively elevated mitochondrial content. Declining bioenergetic fluxes are observed in aging and AD.

Extensive evidence correlates advancing age with declines in overall bioenergetic function (Mecocci et al., 1994; Trifunovic et al., 2004; Kujoth et al., 2005; Navarro and Boveris, 2007; Boveris and Navarro, 2008; Swerdlow, 2011, 2012, 2014; Ross et al., 2013). Decreased glucose utilization via fluorodeoxyglucose (18F) positron emission tomography (FDG-PET) scan analysis is evident in brains from aged individuals (De Santi et al., 1995; Chetelat et al., 2013; Marano et al., 2013). Mitochondria play a prominent role in contributing to the overall state of age-associated bioenergetic decline. Aging mouse models show a continuous decrease in the ability to produce ATP via oxidative phosphorylation at the mitochondrial membrane, and specifically a reduction in the Vmax activities of Complexes I and IV of the electron transport chain (ETC) (Navarro and Boveris, 2007, 2010). Minimal changes to mitochondrial inner membrane permeability or F1-ATP-synthase activity are observed in these aging mouse models. Furthermore, the reduction in Complex I and IV Vmax activities correlates linearly with neurological performance and life span (Navarro and Boveris, 2004, 2007; Boveris and Navarro, 2008). Several studies of rats have shown that inhibiting cytochrome oxidase (COX-IV) results in impaired long term potentiation, further demonstrating the importance of mitochondrial bioenergetic flux in memory and learning (Parker et al., 1990; Bennett et al., 1992; Swerdlow and Kish, 2002). Aging also results in increased reactive oxygen species (ROS) production, a byproduct of dysfunctional mitochondria and oxidative stress (Shigenaga et al., 1994; Ames et al., 1995). Finally, accumulation of somatic mitochondrial DNA (mtDNA) mutations has been implicated as an upstream component in aging phenotypes (Doherty, 2003; Kujoth et al., 2005; Hiona et al., 2010).

Ample evidence indicates age-associated bioenergetic changes are exacerbated in AD populations. Several decades of FDG-PET analyses note a reduction in glucose utilization in AD subject brains (Frackowiak et al., 1981; Foster et al., 1983; Friedland et al., 1983). Analyses of mitochondria isolated from both brain and platelets show an impaired bioenergetic capacity in AD patients. More specifically, there are reductions in Complex I and IV Vmax activity in AD subjects, along with minimal differences in ATP-synthase activity (Mecocci et al., 1994; Maurer et al., 2000; Bosetti et al., 2002). Postmortem examination of AD brain tissue has revealed excessive oxidative stress and oxidative changes to mtDNA (Mecocci et al., 1994; Manczak et al., 2004; Onyango and Khan, 2006; Gibson et al., 2010). Pyruvate dehydrogenase and α-ketoglutarate dehydrogenase are also impaired in AD subjects (Parker et al., 1990; Kish, 1997; Gibson et al., 2010; Swerdlow, 2012).

Although parental history is an AD risk factor, individuals with mothers afflicted with AD are more likely to develop AD than individuals whose fathers have AD (Edland et al., 1996; Mosconi et al., 2007; Honea et al., 2012). Children of mothers with AD have progressive reductions in brain glucose metabolism prior to any overt cognitive defect (Mosconi et al., 2007). Children of cognitively normal parents or fathers afflicted with AD, on the other hand, do not show reductions in brain glucose utilization while phenotypically healthy (Mosconi et al., 2007). As mtDNA is maternally inherited, these data suggest mtDNA may influence AD risk by in part determining an individual’s mitochondrial function and bioenergetic capacity.

The cytoplasmic hybrid (cybrid) technique is a useful way to observe the specific role of mtDNA within disease phenotypes. The technique utilizes the mtDNA contained within donor platelet samples and transfers it to cell lines depleted of endogenous mtDNA. This approach results in the creation of cell lines with an equivalent nuclear DNA background, but that vary in their mtDNA content (Swerdlow, 2002; Wilkins et al., 2014). AD cybrid lines, generated using neuroblastoma cell lines, have decreases in COX activity, ATP, mitochondrial calcium concentration, mitochondrial membrane potential, and glycolytic flux (Sheehan et al., 1997; Swerdlow et al., 1997; Ghosh et al., 1999; Silva et al., 2012, 2013). NFκB, MAPK, and AKT pathways are activated in AD cybrids, while active caspase-3 and cytoplasmic cytochrome *c* are elevated (Ghosh et al., 1999; Onyango et al., 2005; Silva et al., 2012, 2013; Wilkins et al., 2014). Finally, AD cybrids show increased amyloid beta production and mimic the effects of oxidative stress observed in aging and AD brains (Onyango et al., 2005). These cybrid studies indicate the contribution of mtDNA to diminished bioenergetic function and biochemical changes in AD.

It remains to be seen whether bioenergetic dysfunction represents an upstream or downstream pathology in AD. The “mitochondrial cascade hypothesis,” proposed in 2004, asserts that bioenergetic perturbations cause AD clinical and histologic changes (Swerdlow and Khan, 2004). Bioenergetic dysfunction can drive inflammation and, conversely, inflammation can also result in bioenergetic dysfunction. Next, we will review the interplay between these two AD-associated phenomena.

## Relationship between Inflammation and Bioenergetic Dysfunction

Inflammation has the potential to initiate bioenergetic perturbations. Microglia, the resident macrophages within the CNS, function to sense possible pathogen-associated molecular patterns (PAMPs) or damage-associated molecular patterns (DAMPs). Upon activation, microglia produce increased amounts of the reactive nitrogen species (RNS) nitric oxide (NO) due to an up-regulation of inducible nitric oxide synthase (iNOS), and ROS due to an increase in NADPH oxidase (Babior, 2004; Di Filippo et al., 2010). The main function of these free radicals is to mitigate pathogens. However, surrounding cells are also subject to the effects of ROS and RNS. This damages DNA, lipids, and proteins, and can affect overall mitochondrial function. In one possible scheme, free radicals generated by microglia during chronic inflammation states damage mtDNA, which disrupts oxidative phosphorylation and further amplifies ROS/RNS production in a cyclical process (Balaban et al., 2005; Fukui and Moraes, 2008). This is exemplified by the finding that NO production inhibits mitochondrial respiration. The effect is mediated by direct competition of NO against oxygen binding to Complex IV. In particular, astrocytes stimulated to produce NO showed evidence of inhibited mitochondrial respiration (Brown, 1997).

Conversely bioenergetic dysfunction, particularly at the level of the mitochondria, is known to induce inflammation. Inhibition of Complex I with rotenone or methyl-4-phenyl-1,2,3,6-tetrahydropyridine (MPTP) induces inflammatory changes both *in vitro* and *in vivo*. Rotenone administration activates a human microglial cell line and stimulates the production of ROS (Shaikh and Nicholson, 2009). In a rat model, rotenone induces microglial activation in the striatum and substantia nigra. Microglial activation occurs prior to dopaminergic neuron loss in this model (Sherer et al., 2003). A separate study found that rotenone-triggered dopaminergic neuron loss occurs through an NADPH oxidase-dependent microglial response (Gao et al., 2003a). Microglial activation is found in various models of MPTP neurotoxicity. NADPH oxidase, to some extent, is required for MPTP neurotoxicity (Gao et al., 2003b; McGeer and McGeer, 2008; Long-Smith et al., 2009).

Further models associate mitochondrial dysfunction with inflammatory signaling. Inhibition of Complex I (rotenone) or Complex III (antimycin A) in bone marrow-derived macrophages induced IL-1β production, which was dependent on the nod-like receptor family pyrid domain containing 3 (NLRP3) inflammasome (Zhou et al., 2011). In this same study, concomitant rotenone administration with inhibition of mitophagy/autophagy pathways caused an accumulation of damaged mitochondria and ROS production with downstream IL-1β production (Zhou et al., 2011). Macrophages lacking mtDNA (ρ0 cells) are not able to activate caspase-1 in response to LPS and ATP (Nakahira et al., 2011). This study indicates the requirement for functional mitochondrial respiration in the activation of caspase-1 and the NLRP3 inflammasome.

A further study utilized the cybrid model to generate cell lines with mtDNA from different haplogroups. mtDNA haplogroups represent distinct population origins through defined SNPs. This particular study compared the H haplogroup to the J haplogroup. The J haplogroup had significantly decreased expression of seven mitochondrial-encoded ETC components (MT-ND1, MT-ND2, MT-ND3, MT-ND4, MT-CO2, MT-CO3, and MT-ATP6) (Cristina Kenney et al., 2014). Cybrids generated from J haplogroup mtDNA donors had a reduced oxygen consumption rate (OCR), a reduced OCR to extracellular acidification rate (ECAR) ratio, and significantly reduced complement pathway and other inflammatory gene mRNA levels (such as IL-33). This novel study depicts the interplay between specific mtDNA sequences, bioenergetic function, and inflammation.

It is increasingly apparent that bioenergetic function and inflammation are interdependent processes. We further hypothesize the release of DAMPs that derive from mitochondria may at least, in part, drive inflammation in AD.

## Sterile Inflammation

Given the striking lack of evidence for pathogen-induced inflammation in AD, it is reasonable to hypothesize a significant role for non-pathogen initiated inflammation or sterile inflammation in the disease pathology. DAMPs, also referred to as alarmins, are endogenous molecules that are normally sequestered by the host cell and are therefore recognized as danger signals. DAMPs are released during the death and rupture of host cells. These molecules initiate inflammatory signaling cascades, thus leading to sterile inflammation. DAMPs have been associated with traumatic brain injury, ischemia-reperfusion, atherosclerosis, arthritis, and systemic inflammatory response syndrome (SIRS) (Collins et al., 2004; Pullerits et al., 2005; Porto et al., 2006; Foell et al., 2007; Zhang et al., 2010; van Golen et al., 2013; Walko et al., 2014).

**Figure 1:**
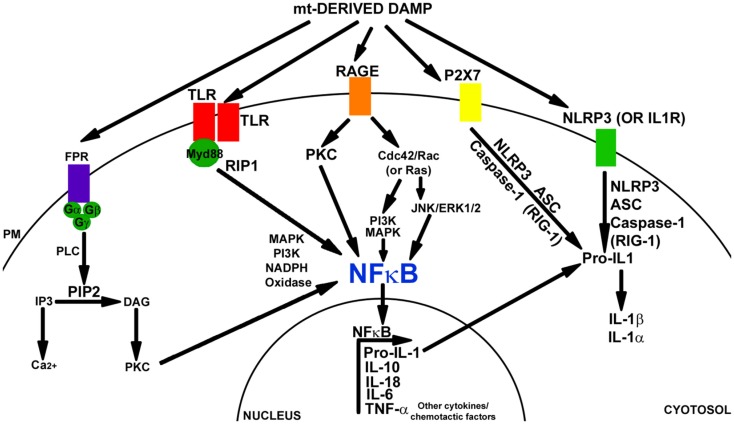
**Damage-associated molecular pattern signaling mechanisms**. Mitochondrial components can induce a DAMP response through the activation of TLR, FPR, RAGE, P2X7, and/or the NALP3 inflammasome. FPR is a GCPR. FPR signaling is mediated through PLC, PIP2, IP3, and DAG. Downstream activation of PKC leads to signaling through MAPK, PI3K, and activation of NFκB and NAPDH oxidase. TLR receptors dimerize with Myd88 and can activate NFκB through RIP1 kinase, MAPK, or PI3K pathways. The TLR pathway also activates NADPH oxidase. RAGE receptor signaling activates PKC and CdC42/Rac (or Ras). Downstream PI3K, MAPK or JNK, and Erk1/2 are activated. This leads to NFκB activation (in addition to SP1 and AP1). P2X7 activation leads to NRLP3 inflammasome signaling. NLRP3 (or IL-1R) activation mediates inflammasome signaling through NLRP3, ACS, and caspase-1 (or RIG-1). Pro-IL-1 is cleaved into IL-1β and IL-1α. NFκB activation initiates transcription of Pro-IL-1, IL-10, IL-18, IL-6, TNFα, along with many other cytokine and chemotactic factors.

The inflammatory response stimulated by DAMPs is similar to the response induced by PAMPs. Typically, these molecules are recognized by a pattern recognition receptor (PRR), which leads to the activation of transcription factors such as NFκB and cytokine production. Despite this similarity, data suggest PRRs are able to discriminate between PAMPs and DAMPs through CD24-SiglacG/10 signaling (Kaczmarek et al., 2013). Figure 1 depicts the pathways in which DAMP molecules can initiate inflammatory signaling.

### Mitochondrial components as DAMPs

Mitochondria presumably arose through the modification of a previously independent, respiratory competent prokaryotic cell. This is supported by the fact that mitochondria share numerous characteristics with bacteria. Several studies have examined the ability of whole mitochondrial extracts to induce inflammation. In particular, human plasmacytoid dendritic cells incubated with total mitochondrial extracts up-regulate IFNα expression (Julian et al., 2012). This requires the presence of mtDNA and toll-like receptor 9 (TLR9)/receptor for advanced glycation endproducts (RAGE) activation. Fractions from nuclear and cytoplasmic pools, on the other hand, do not induce IFNα expression. A separate study that treated human blood monocytes with total mitochondrial extracts found increased IL-8 expression and secretion (Crouser et al., 2009). Again, treatment with nuclear or cytoplasmic fractions did not produce this result. *In vivo* and *in vitro* treatment with disrupted mitochondria induced polymorphonuclear leukocyte (PMN) migration and degranulation, calcium flux, MAPK activation, and cytokine production that included increased levels of matrix metalloproteinase-8 (MMP-8), IL-8, IL-6, and TNFα (Zhang et al., 2010). Human neutrophils treated with ruptured mitochondria showed increased calcium release, oxidative bursts, and chemotaxis (Julian et al., 2013). Overall, it is not surprising that a considerable number of molecules that derive from mitochondria act as DAMPs. These molecules are shown in Table 2 and are reviewed below.

**Table 2:** **Mitochondrial-derived DAMP molecules**.

Mitochondrial derived-DAMP molecule	Receptor activated	Proteins activated/up-regulated	Cytokines produced	Reference
mtDNA	TLR	MAPK, MMP-8, NFκB	IL-1β, IL-6, IL-8, MCP-1, TNFα	(Oka et al., 2012; Zhang et al., 2014)
Cardiolipin	NA	ICAM, VCAM	NA	(Wan et al., 2014)
ATP	P2X7, NLRP3	MMP9	CCL2, CCL7, CXCL2, IL-1β, IL-6, IL-8, IL-10, IL-12, IL-18, INFγ, TNFα	(Gourine et al., 2007; Piccini et al., 2008; Riteau et al., 2010; Kurashima et al., 2012; Cauwels et al., 2014)
fMLP	FPR	iNOS, NFκB, MAPK, PI3K	IL-1β, IL-8	(Pan et al., 2000)
TFAM+/−mtDNA	TLR, RAGE	NFκB, PI3K	IL-1β, IL-6, IL-8, TNFα	(Julian et al., 2013; Little et al., 2014)
Cytochrome c	NA	NFκB	IL-6, MCP-1, MIP-2, MIP-1α RANTES, TNFα	(Pullerits et al., 2005)
HMGB1	TLR, RAGE	ERK, ICAM, JNK, NFκB, MAPK, SP1, VCAM	IL-8, MCP-1, PAI-1, tPA, TNFα	(Scaffidi et al., 2002; Fiuza et al., 2003; Mazarati et al., 2011)

*NA, not available*.

#### Mitochondrial DNA

Mitochondrial DNA shares many characteristics with bacterial DNA. It is circular in structure, lacks histones, and contains unmethylated CpG repeats and bound formyl-peptides. The mitochondrial genome encodes 11 components of the ETC and two subunits of ATP synthase. The nuclear genome and mitochondrial genome work in an organized and concerted manner to maintain a functional ETC. mtDNA serves as a DAMP molecule in trauma patients and in SIRS. In particular, the concentration of mtDNA circulating in plasma predicts mortality in trauma patients (Zhang et al., 2010; Nakahira et al., 2013).

Several studies have directly tested the ability of mtDNA to induce inflammation. In a recent study, mouse primary astrocytes were transfected with oxidant-initiated, degraded mitochondrial polynucleotides (DeMPs) (Mathew et al., 2012). This lead to the production of IL-6, MCP-1, TNFα, and IL-1β. DeMPs can also be detected in human CSF samples. In human PMNs, mtDNA induces MAPK activation and IL-8 production (Zhang et al., 2010). mtDNA also induces inflammation *in vivo*. Intra-articular injection of mtDNA in mice induces arthritis and TNFα expression (Collins et al., 2004). The lungs of rats receiving intravenous injection of mtDNA exhibited up-regulation of TLR9, NFκB, TNFα, IL-6, and IL-10 (Zhang et al., 2014). These results were not recapitulated upon injection of nuclear DNA. A separate study demonstrated mtDNA that eludes autophagy can induce inflammation (Oka et al., 2012). Here, mice with cardiac-specific deletion of DNAse II underwent pressure overload, which allowed the release of mtDNA while subsequently inhibiting its degradation. Increased IL-1β and IL-6 were observed. Outcomes in these mice were improved following the genetic ablation of TLR9. Overall, in various cell and tissue types mtDNA induces inflammatory signaling and cytokine production through common mechanisms.

#### Mitochondrial transcription factor A

Mitochondrial transcription factor A (TFAM) binds to mtDNA to initiate its transcription. Beyond its function in transcription of mtDNA, TFAM can also be released from damaged and dying cells, where it can act as a DAMP molecule. Specifically, treatment of human peripheral blood monocytes, THP-1 monocytic cells, or primary microglia from autopsy patients with TFAM and IFNγ caused cell death in neuronal cell lines co-cultured with glial cells. It was found that activation of the JNK pathway was necessary for these results (Little et al., 2014). Stimulation of THP-1 monocytes with TFAM alone elevated expression of IL-1β, IL-6, and IL-8, and this was enhanced by the addition of IFNγ. An additional study found that TFAM alone or in combination with formyl-peptides induced IL-8 release from human blood monocytes in a formyl peptide receptor (FPR)-dependent manner (Crouser et al., 2009).

The combination of TFAM bound to mtDNA can also induce inflammation (Julian et al., 2012, 2013). This was demonstrated in human plasmacytoid dendritic cells, which up-regulated IFNα expression and release upon exposure to TFAM-bound mtDNA. In this study, inhibition of RAGE and TLR9 inhibited IFNα expression. PI3K, ERK, and NFκB signaling were also implicated.

Mouse splenocytes treated with a combination of TFAM and CpG-enriched mtDNA released TNFα in a TLR9 and RAGE-dependent manner (Julian et al., 2013). PI3K and NFκB were also required for TNFα expression. Thus, TFAM can induce inflammation either alone or in combination with other mitochondrial components.

#### Cardiolipin

Mitochondrial lipids also have prokaryotic features. Cardiolipin is a unique lipid found in bacteria and the mitochondrial inner membrane. Cardiolipin is essential to mitochondrial function. Cardiolipin also acts as a mitochondrial-derived DAMP molecule. Anti-cardiolipin antibodies are found in some autoimmune diseases, including lupus (Ishii et al., 1990). Further, treatment of human monocyte-derived macrophages with oxidized cardiolipin induced the expression of intracellular adhesion molecule (ICAM-1, also known as CD54) and vascular cell adhesion molecule (VCAM-1, also known as CD106) (Wan et al., 2014). Non-oxidized cardiolipin failed to elicit similar responses. While cardiolipin has the potential to induce inflammation and act as a DAMP molecule, at this time there is little direct evidence to suggest it commonly functions in this capacity.

#### Cytochrome *c*

Cytochrome *c* is a small protein that is tethered by cardiolipin to the inner mitochondrial membrane. It serves as an electron donor and acceptor during oxidative phosphorylation. The release of cytochrome *c* from mitochondria induces apoptosis, while its extracellular release initiates a DAMP response. Intra-articular injection of cytochrome *c* in mice induces arthritis (Pullerits et al., 2005). In addition, exposing mouse splenocytes to exogenous cytochrome *c* activates NFκB as well as TNFα, IL-6, macrophage inflammatory proteins (MIP-2α or CXCL2; MIP-1α or CCL3), MCP-1, and RANTES (regulated on activation, normal T cell expressed) production. Circulating cytochrome *c* can be measured in patients with liver injury, SIRS, and myocardial infarction (Krysko et al., 2011). Similar to cardiolipin, antibodies against cytochrome *c* are found in lupus patients (Mamula et al., 1990).

#### Adenosine triphosphate

The main function of mitochondria in many cell types is to produce ATP through oxidative phosphorylation. A release of ATP occurs during cell death through either an active mechanism prior to loss of cell membrane integrity, or via a passive process after cell membranes become permeable (Zitvogel et al., 2010). ATP facilitates the recruitment of macrophages and activation of the NLRP3 inflammasome through P2X7 purinergic receptors. In a mouse model of 2,4,6-trinitrobenzene sulfonic acid (TNBS) induced-colitis, genetic knockout of P2X7 in mast cells reduced intestinal inflammation and IL-6, TNFα, MCP-1, MCP-3, and MIP-2α cytokine production (Kurashima et al., 2012). Inhibition of ADP-responsive P2Y receptors (P2Y1 and P2Y12) had no effect on inflammation observed in this model.

Using LPS-induced shock in mice, a separate study demonstrated a requirement for extracellular ATP in inflammation (Cauwels et al., 2014). This study used an ATP degrading enzyme, apyrase, to show extracellular release of ATP is required for inflammation and IL-1β, TNFα, and IL-10 cytokine production. In this study, genetic ablation of P2X7 receptors also mitigated cytokine production (Riteau et al., 2010). Conversely, when ATP-γS (a stable ATP derivate) was administered, inflammation was enhanced.

Within the CNS, extracellular ATP can serve as a DAMP molecule and also plays a role in the regulation of body temperature, cardiovascular function, and respiratory control (Gourine et al., 2007). As a DAMP molecule, ATP initiates TNFα release from cultured rat microglia in a calcium-dependent manner (Hide et al., 2000). Extracellular ATP treatment of microglia enhanced expression of MAPK and ERK, and inhibition of these pathways prevented production of TNFα. Primary rat cortical astrocytes treated with LPS release TNFα, while ATP treatment alone has no effect (Kucher and Neary, 2005).

*In vivo* injection of LPS into rat striatum activates P2X7 receptors (Choi et al., 2007). Blocking these receptors inhibits MAPK and NFκB activation, and inhibits the production of COX2, IL-1β, IL-6, IL-12, and TNFα. Other studies have shown a differential effect of ATP on inflammation. ATP-induced TNFα release in microglia cells was found to protect co-cultured neurons exposed to glutamate (Suzuki et al., 2004).

In one study, injection of LPS into the anterior hypothalamus of rabbits induced an extracellular ATP release (Gourine et al., 2007). The concentration and timing of this extracellular ATP release appeared to associate with a thermoregulatory febrile response (Gourine et al., 2007).

#### Formyl-peptides

Formyl-peptides are found in both mitochondrial compartments and bacteria. In particular, formyl-methionine–leucine–phenylalanine (fMLP) is known to act as a DAMP molecule. In human peripheral blood monocytes, fMLP induces NFκB activation and IL-1β production in a PI3K-dependent manner (Pan et al., 2000). Similar results were observed in murine peritoneal macrophages. fMLP stimulation led to activation of NFκB, PI3K, and MAPK pathways. Furthermore, iNOS expression and NO production were increased, an effect that depended on L-arginine (Sodhi and Biswas, 2002).

In PMN cells, fMLP results in calcium release and chemotaxis (Raoof et al., 2010). fMLP also induces inflammation in microglial cultures. Mixed cultures of neurons and glia stimulated with fMLP showed selective dopaminergic neuron loss and decreased dopamine uptake (Gao et al., 2008). Pharmacologic inhibition or genetic ablation of NADPH oxidase blocked fMLP toxicity. fMLP-related microglial activation was associated with changes in morphology, MHC II expression, and extracellular superoxide production. Formyl-peptides, specifically those with the sequence fMLP, therefore, initiate inflammation in peripheral and CNS cell models.

#### High mobility group box 1

High mobility group box 1 is normally considered a nuclear protein, however, under both normal and pathological conditions it localizes to mitochondria (Stumbo et al., 2008; Tang et al., 2010). The oxidation state of high mobility group box 1 (HMGB1) may influence its cellular localization (Tang et al., 2010). In the nucleus, HMGB1 functions to regulate transcription through interactions with histones and transcription factors. It also functions as a cytokine. In mitochondria, HMGB1 regulates mitochondrial “quality control” (Tang et al., 2010). Immune cells (such as macrophages) actively secrete HMGB1 during inflammation (Andersson and Tracey, 2011). Recent studies have also depicted the DAMP function of HMGB1. In mice, intracerebroventricular injection of HMGB1 caused memory deficits through TLR4 and RAGE-dependent pathways (Mazarati et al., 2011).

High mobility group box 1 also mediates inflammation *in vitro*. Necrotic mouse fibroblast cells release HMGB1, stimulating inflammatory changes such as increased TNFα production via NFkB (Scaffidi et al., 2002). Indeed, necrotic fibroblast cells derived from HMGB1 null mice are not capable of inducing inflammation. Exogenous incubation of human microvascular endothelium cells with HMGB1 induces expression of ICAM-1, VCAM-1, RAGE, TNFα, IL-8, MCP-1, plasminogen activator inhibitor-1 (PAI-1), and tissue plasminogen activator (tPA) (Fiuza et al., 2003). Cell signaling pathways and transcription factors that are activated include MAPK, ERK, JNK, NFκB, and SP1.

### Mechanisms for mitochondrial-derived DAMP release

Specific mitochondrial components clearly act as DAMP molecules. Beyond this, total mitochondrial extracts induce inflammation in several cell types (Crouser et al., 2009; Zhang et al., 2010; Julian et al., 2012, 2013). There is mounting evidence in the literature supporting mechanisms in which mitochondrial components (either as part of whole mitochondria or as specific molecules) can be released from cells. For example, mitochondria that escape autophagy (specifically, mitophagy) have the potential to stimulate an inflammatory response (Oka et al., 2012). A recent study showed that retinal ganglion cells shed mitochondria at the optic nerve head (Davis et al., 2014). Mitochondria shed by these neurons are then internalized by surrounding astrocytes and degraded. The authors of the study referred to this process as transmitophagy, or transcellular degradation of mitochondria. This process is also suspected to occur in other parts of the CNS, as similar accumulations of degrading mitochondria are found in superficial layers of the cerebral cortex alongside neurites. Finally, extracellular release of mitochondrial components could be a consequence of cell death pathways, such as necrosis or, possibly necroptosis (Kaczmarek et al., 2013).

### Evidence for mitochondrial-derived DAMPs in AD pathology

As discussed above, several mitochondrial components can act as DAMP molecules and evoke inflammatory signaling cascades. Here, we discuss relevant data, which suggest a role for mitochondrial DAMPs in AD pathology.

#### DAMP signaling in AD

Inflammasomes, particularly NLRP3 (or NALP3), have recently generated interest in the AD research field (Shaftel et al., 2008). Several components comprise the NLRP3 inflammasome including NLR protein, ASC adaptor protein, and pro-caspase-1. Retinoic acid inducible gene-1 (RIG-1) is another potential component of the inflammasome. Upon activation, the NLR protein activates transcription of pro-IL-1β and the formation of the NLRP3 inflammasome complex. After this, the NLRP3 inflammasome cleaves and activates pro-IL-1β and downstream molecules can be activated. Inflammasome signaling has been extensively reviewed elsewhere (Latz et al., 2013). Current data suggest IL-1β polymorphisms may associate with AD risk, although caspase-1 polymorphisms do not (Griffin et al., 2000; McGeer and McGeer, 2001; Shaftel et al., 2008; Vazquez-Higuera et al., 2010).

IL-1β levels are increased in CSF and serum from AD patients (Cacabelos et al., 1991; Blum-Degen et al., 1995; Licastro et al., 2000). Furthermore, IL-1 is found in microglia surrounding plaques in postmortem AD brain tissue (Griffin et al., 1989). IL-1β experimentally induces the disruption of the BBB and leukocyte recruitment to the CNS while also blocking LTP in the hippocampus (Shaftel et al., 2008). Elevated expression of active caspase-1 has also been detected in AD brain tissue (Heneka et al., 2013). RIG-1, a component of inflammasome signaling, is elevated in plasma and temporal cortex in mild cognitive impairment (MCI, frequently a precursor syndrome of AD) subjects (de Rivero Vaccari et al., 2014) Therefore, many lines of evidence suggest the NLRP3 inflammasome is relevant to AD.

Toll like receptors may also play a pivotal role in AD. Specific TLR9 polymorphisms have been associated with a decreased AD risk, while specific TLR4 polymorphisms have been associated with an increased risk (Balistreri et al., 2008; Wang et al., 2011, 2013). There is some conflict in the literature regarding the association of TLR2 genetic polymorphisms in AD (Yu et al., 2011a,b). In blood (PBMCs specifically) and brain tissue from AD patients, TLR2 and TLR4 are increased (Liu et al., 2005; Walter et al., 2007; Letiembre et al., 2009; Zhang et al., 2012). TLR activation leads to receptor dimerization at the plasma membrane with the adaptor protein MyD88, activation of the RIP1 kinase, and downstream signaling to NFκB and MAPK. Changes in levels of IL-6, IL-8, IL-12, and TNFα have been reported. Similar changes are observed in AD (Singh and Guthikonda, 1997; Swardfager et al., 2010; Cojocaru et al., 2011; Alsadany et al., 2013). While a lack of data exists regarding MyD88 in human AD patients, in one AD mouse model genetic ablation of MyD88 was found to mitigate microglial activation and amyloid beta toxicity (Lim et al., 2011).

Formyl peptide receptors are seven transmembrane domain-configured G protein-coupled receptors (GPCRs). Activation of FPRs can induce cell adhesion, chemotaxis, ROS release, production of pro-inflammatory cytokines, and phagocytosis. Upon stimulation, FPRs bind G proteins and execute the activation of signaling pathways including, PI3K, IPR3 (which mediates ER calcium efflux), and NFκB. This leads to downstream NADPH oxidase respiratory flux and cytokine production. FPRs have attracted interest in AD due to the binding capacity of the amyloid beta peptide (Verdier et al., 2004). While the downstream effectors of FPR signaling are implicated in AD histopathology, no direct evidence of activation of FPRs in AD patients has currently been published.

RAGE was initially identified in lung tissue, where it was found to bind advanced glycation endproducts (AGEs) (Xie et al., 2013). Numerous cell processes are activated through RAGE signaling including cell motility, proliferation, autophagy, inflammation, and apoptosis. Inflammatory signaling cascades activated downstream of RAGE include MAPK, JNK, and NFκB. Production of pro-inflammatory cytokines, including IL-6, IL-8, TNFα, and COX2, are mediated through these signaling cascades. Several mitochondrial-derived DAMPs can induce RAGE activation, including the combination of TFAM and CpG mtDNA (Julian et al., 2013). RAGE also binds amyloid beta, a finding that suggests a possible association between RAGE and AD (Verdier et al., 2004; Xie et al., 2013). Microglia from AD subjects show increased RAGE expression (Lue et al., 2001).

#### Mitochondrial-derived DAMPs in AD

Few mitochondrial-derived DAMP molecules have been measured in actual AD patients. CSF concentrations of cell-free circulating mtDNA are reduced in AD patients (Podlesniy et al., 2013). This finding is of interest because while amyloid beta levels are high in AD brain and plasma, CSF levels of amyloid beta are decreased (Mehta et al., 2000; Wallin et al., 2006; Buchhave et al., 2012). How CSF concentrations of molecules (such as proteins and DNA) relate to their respective brain concentrations at this time, therefore, is incompletely understood. Another study that reported DeMPs induce inflammation in primary mouse astrocytes (Mathew et al., 2012) also reported the detection of DeMPs in human CSF and plasma samples. However, no current data are available to show whether DeMP levels in AD subjects exceeds levels in non-demented control subjects.

Cerebral spinal fluid cytochrome *c* levels are increased in MCI patients, and to some extent can predict conversion to AD. More specifically, cytochrome *c* levels are increased in MCI subjects who ultimately convert to the AD clinical phenotype (Papaliagkas et al., 2009).

## Conclusion

Inflammation and bioenergetic dysfunction are important pathological phenomena in AD. Inflammation and bioenergetic dysfunction can exhibit a cyclical relationship. Given the relationship between these two pathologies and their interdependence on mitochondria we hypothesize that DAMPs derived from mitochondria contribute to the initiation of inflammation in AD. The release of mitochondrial components could occur through necrotic cell death, transcellular mitophagy, or other currently undefined mechanisms. The presence of inflammation may, in turn, further perturb brain bioenergetic function. Basic evidence for a DAMP response in AD is currently supported by existing literature, although further investigation is needed to specifically define the contribution of mitochondrial DAMPs to chronic brain inflammation in this disease.

## Conflict of Interest Statement

The authors declare that the research was conducted in the absence of any commercial or financial relationships that could be construed as a potential conflict of interest.

## Abbreviations

ABCA7, ATP-binding cassette, sub-family A (ABC1), member 7; AD, Alzheimer’s disease; AGE, advanced glycation endproduct; AKT, protein kinase B; ATP, adenosine triphosphate; CCR3, C-C motif receptor 3; CCR5, C-C motif receptor 5; CD33, cluster of differentiation 33; CLU, clusterin; CNS, central nervous system; COX2, cyclooxygenase 2; COX-IV, cytochrome oxidase; CpG, cytosine followed by guanine cite of methylation in DNA; CR1, complement receptor 1; CRP, C reactive protein; CSF, cerebral spinal fluid; CXCR2, chemokine C-X-C motif receptor 2; DAMP, damage-associated molecular pattern; DeMP, oxidant-initiated degraded mitochondrial polynucleotides; ECAR, extracellular acidification rate; EPHA1, epherin receptor A1; ERK, extracellular signal-regulated kinase; ETC, electron transport chain; FDG-PET, fluorodeoxyglucose (18F) positron emission tomography; fMLP, formyl-methionine, leucine, phenylalanine; FPR, formyl peptide receptor; GPCR, G protein-coupled receptor; GWAS, genome wide association study; HMGB1, high mobility group box 1; ICAM, intracellular adhesion molecule; IFNα, interferon alpha; IL-1, interleukin 1; IL-6, interleukin 6; IL-8, interleukin 8; IL-10, interleukin 10; IL-12, interleukin 12; INFγ, interferon gamma; iNOS, inducible nitric oxide synthase; INPPD5, inositol polyphosphate-5-phosphatase; JNK, c-Jun N-terminal kinase; LPS, lipopolysaccharide; MAC, membrane attack complex; MAPK, mitogen activated protein kinase; MCI, mild cognitive impairment; MCP-1, monocyte chemotactic protein 1; MCP-3, monocyte chemotactic protein 3; MEFC2, myocyte-specific enhancer factor 2C; MHCII, major histocompatibility class II; MIP-1α, macrophage inflammatory protein 1 alpha; MIP-2α, macrophage inflammatory protein 2 alpha; MMP-8, matrix metalloproteinase-8; MPTP, methyl-4-phenyl-1,2,3,6-tetrahydropyridine; mtDNA, mitochondrial DNA; NFκB, nuclear factor kappa-light-chain-enhancer of activated B cells; NLRP3, NOD-like receptor family, pyrin domain containing 3; NO, nitric oxide; NSAID, non-steroidal anti-inflammatory drug; OCR, oxygen consumption rate; PAI-1, plasminogen activator inhibitor-1; PAMP, pathogen-associate molecular pattern; PI3K, phosphatidylinositol-4,5-bisphosphate 3-kinase; PRR, pattern recognition receptor; PTK2B, protein tyrosine kinase 2 beta; RAGE, receptor for advanced glycation endproducts; RANTES, regulated on activation, normal T cell expressed and secreted; RIG-1, retinoic acid inducible gene-1; RNS, reactive nitrogen species; ROS, reactive oxygen species; SIRS, systemic inflammatory response syndrome; SNP, single nucleotide polymorphism; TCA, citric acid cycle; TFAM, mitochondrial transcription factor A; TGFβ, transforming growth factor beta; TLR, toll like receptor; TNBS, 2,4,6-trinitrobenzenesulfonic acid; TNFα, tumor necrosis factor alpha; tPA, tissue plasminogen activator; TREM2, triggering receptor expresses on myeloid cells 2; VCAM, vascular cell adhesion molecule.
